# The effect of glycosylated hemoglobin levels on the response to intravitreal dexamethasone implant for treating diabetic macular edema

**DOI:** 10.1038/s41598-024-55078-6

**Published:** 2024-02-26

**Authors:** Hyuk Jun Lee, Kunho Bae, Chang Ki Yoon, Un Chul Park, Kyu Hyung Park, Eun Kyoung Lee

**Affiliations:** Department of Ophthalmology, Seoul National University College of Medicine, Seoul National University Hospital, #101, Daehak-ro, Jongno-gu, 03080 Seoul, Republic of Korea

**Keywords:** Outcomes research, Retinal diseases

## Abstract

This study investigates the impact of glycosylated hemoglobin (HbA1c) on the efficacy of intravitreal dexamethasone (DEX) implants in patients with diabetic macular edema (DME) over a 12-month period. We retrospectively reviewed 90 DME patients treated with DEX implants, categorizing them based on baseline HbA1c levels (≤ 7% and > 7%) and 12-month changes in HbA1c ("improved", "stable", "worsened"). At the 2-month mark, the mean central subfield thickness (CST) reduction in the HbA1c ≤ 7% group was − 147.22 ± 113.79 µm compared to -130.41 ± 124.50 µm in the > 7% group (*p* = 0.506). Notably, 12-month outcomes between these groups showed no significant difference. The "improved" HbA1c subgroup experienced a more pronounced CST reduction at 2 months (*p* = 0.042), with outcomes leveling off with other groups by 12 months. Conclusively, DEX implant outcomes in DME were not influenced by either baseline HbA1c levels or their changes over time. This suggests that local alterations in the inflammation milieu may have a potentially stronger impact on DME treatment outcomes, highlighting the importance of considering local factors in DME treatment.

## Introduction

Diabetic macular edema (DME) is a leading cause of visual impairment in patients with diabetes mellitus. DME occurs when the blood-retina barrier is disrupted, causing vascular leakage and fluid accumulation in the macula^1^. The global prevalence of DME is estimated to be 7.5%, affecting approximately 21 million individuals worldwide^2^. Inflammatory and angiogenic components play a central role in the pathogenesis of DME^3^. Since the advent of anti-vascular endothelial growth factor (anti-VEGF) agents for treating patients with DME, anatomical improvements represented by macular thickness reduction and a significant improvement in visual outcome have been demonstrated^4^. However, more effective therapies with longer durability are needed for patients with DME who do not respond adequately to anti-VEGF therapy^5–7^. Dexamethasone implants (DEX implants, Ozurdex®, Allergan Inc., Irvine, CA, USA) has become a good therapeutic alternative for DME since they meet this need with favorable outcomes^8^.

Several epidemiological studies have revealed that elevated glycosylated hemoglobin (HbA1c) levels increase DME risk^2,9,10^. However, once patients develop DME, it is unclear whether glycemic control affects their response to treatment. Patients’ variable response to DME treatment may be influenced by systemic factors including the duration of diabetes, serum HbA1c levels, hypertension, and nephropathy. Several studies have investigated the influence of HbA1c on the efficacy of anti-VEGF therapy for treating DME, with inconclusive results. However, to the best of our knowledge, there have been very few reports on the influence of HbA1c levels on the effect of DEX implants in the eyes with DME.

This study aimed to investigate the influence of serum baseline HbA1c and changes in HbA1c during the study on treatment outcomes in patients with DME treated with intravitreal DEX implants. The need for retreatment was also evaluated.

## Results

### Baseline characteristics: baseline HbA1c analysis

This study included 90 eyes of 90 patients. According to the study classification criteria, 41 (45.6%) eyes were included in patients with baseline HbA1c ≤ 7% and 49 (54.4%) were included in those with baseline HbA1c > 7%. The patient demographics and baseline characteristics are summarized in Table 1. The mean baseline HbA1c level was 6.33 ± 0.48% in patients with baseline HbA1c ≤ 7% (range, 5.3 − 6.9%) and 7.87 ± 0.78% in those with baseline HbA1c > 7% (range, 7.0 − 10.1%; *p* < 0.001). Patients with baseline HbA1c > 7% were significantly older (mean age, 67.22 ± 7.53 years), had a longer diabetes mellitus (DM) duration (21.27 ± 9.02 years), and were more likely to be women (28/49, 57.1%) than those with baseline HbA1c ≤ 7% (mean age 61.29 ± 9.90 years, *p* = 0.002; 12.73 ± 7.74 years, *p* < 0.001; women 13/41, 31.7%, *p* = 0.028). There was no significant difference in the proportion of patients taking Sodium-glucose co-transporter 2 inhibitors (SGLT2i) between subgroups (*p* = 0.347). The baseline lens status was not significantly different between the two subgroups (*p* = 0.531). The mean baseline best-corrected visual acuity (BCVA), central subfield thickness (CST), diabetic retinopathy (DR) grading, and previous treatment for DME were not significantly different between the two subgroups (*p* = 0.368, *p* = 0.727, *p* = 0.142, and *p* = 0.228, respectively).

**Table 1:** Patient demographics, baseline characteristics, and summary of retreatment: Baseline glycosylated hemoglobin analysis.

Variable	Baseline HbA1c ≤ 7%	Baseline HbA1c > 7%	*p* value
Number of eyes	41 (45.6%)	49 (54.4%)	
HbA1c (%)	6.33 ± 0.48	7.87 ± 0.78	< 0.001*
DM duration (years)	12.73 ± 7.74	21.27 ± 9.02	< 0.001*
Use of SGLT2i	2 (4.9%)	5 (10.2%)	0.347†
Age (years)	61.29 ± 9.90	67.22 ± 7.53	0.002*
Men/Women	28/13	21/28	**0.028†**
RE/LE	27/14	21/28	**0.049†**
Phakia/Pseudophakia	27/14	28/21	0.531†
BCVA (logMAR)	0.48 ± 0.33	0.54 ± 0.27	0.368*
CST (µm)	447.24 ± 142.77	437.37 ± 120.65	0.727*
DR grade			0.142†
Moderate NPDR	2 (4.9%)	6 (12.2%)	
Severe NPDR	10 (24.4%)	18 (36.7%)	
PDR	29 (70.7%)	25 (51.0%)	
Previous treatment for DME			0.228†
Treatment of naïve	19 (46.3%)	15 (30.6%)	
Focal/grid laser	3 (7.3%)	8 (16.3%)	
Anti-VEGF	19 (46.3%)	21 (42.9%)	
Triamcinolone	5 (12.1%)	11 (22.4%)	
Time to retreatment	4.34 ± 4.56	6.04 ± 3.82	0.062*
Number of retreatments	1.02 ± 1.15	1.02 ± 0.80	0.985*
Retreatment for DME			0.108†
Anti-VEGF	18 (43.9%)	11 (22.4%)	
DEX implant	15 (36.6%)	26 (53.1%)	
Triamcinolone	4 (9.8%)	5 (10.2%)	
Adverse event			
Cataract	8 (19.5%)	6 (12.2%)	0.343†
Increased IOP	2 (4.9%)	1 (2.0%)	0.455†
Endophthalmitis	0	0	
Retinal detachment	0	0	

HbA1c = glycosylated hemoglobin; DM = diabetes mellitus; RE = right eye; LE = left eye; BCVA = best-corrected visual acuity; logMAR = logarithm of the minimum angle of resolution; CST = central subfield thickness; DR = diabetic retinopathy; NPDR = nonproliferative diabetic retinopathy; PDR = proliferative retinopathy; DME = diabetic macular edema; VEGF = vascular endothelial growth factor; DEX = dexamethasone; IO* p* = intraocular pressure; SGLT2i = Sodium-glucose co-transporter 2 inhibitor.

*Student’s *t*-test. †Chi-square test.

Continuous variables are reported as mean ± standard deviation. All other data are numbers (percentages). Significant values with *p* < 0.05 are in bold.

### Baseline HbA1c and clinical outcomes

**Figure 1:**
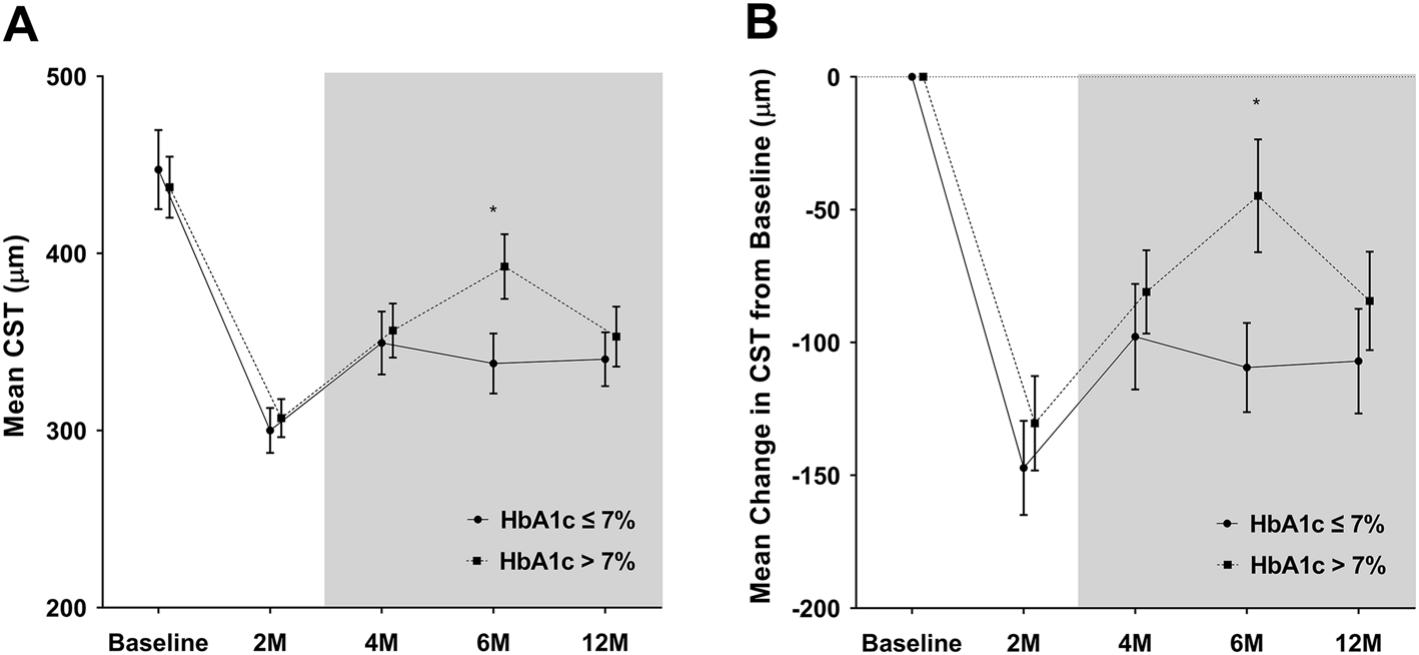
The mean CST (**A**) and the mean change in CST from baseline (**B**) through the follow-up periods in DEX implant-treated eyes with baseline HbA1c ≤ 7% compared with HbA1c > 7%. Circles and squares indicate means, and error bars represent ± 1 standard error. The gray shaded area is the time when retreatment was permitted. Asterisks denote significant difference (*p < 0.05, ** p < 0.01) between the two groups at each follow-up visit. CST = central subfield thickness; HbA1c = glycosylated hemoglobin.

The anatomical and visual outcomes following the baseline HbA1c levels are summarized in Table 2. The mean CST was significantly reduced at 2 months after DEX implant injection; however, there was a gradual increase at 4 and 6 months in both groups (Fig. 1). At 2 months, when the pure effect of DEX implant was expected, the mean CST reduction from baseline was − 147.22 ± 113.79 µm in patients with baseline HbA1c ≤ 7% and − 130.41 ± 124.50 µm in patients with baseline HbA1c > 7%, which was not significantly different (*p* = 0.506). At 6 months, when retreatment was implemented, the mean CST in patients with baseline HbA1c > 7% (392.59 ± 128.02 µm) was significantly higher than that in patients with baseline HbA1c ≤ 7% (337.80 ± 108.42 µm, *p* = 0.031). However, the mean CST and CST reduction at 12 months was not significantly different between the two groups (*p* = 0.577 and *p* = 0.405, respectively).

**Table 2:** Serial changes in mean CST, mean CST change, mean BCVA, and mean BCVA change were compared between eyes with baseline HbA1c ≤ 7% and those with baseline HbA1c > 7%.

Variable	Eyes	Baseline	2 months	4 months	6 months	12 months
CST (µm)	HbA1c ≤ 7%	447.24 ± 142.77	300.02 ± 81.34	349.44 ± 113.96	337.80 ± 108.42	340.27 ± 97.70
	HbA1c > 7%	437.37 ± 120.65	306.96 ± 75.68	356.41 ± 107.44	392.59 ± 128.02	353.02 ± 118.57
	*p* value	0.727	0.679	0.768	**0.031**	0.577
Change in CST from baseline (µm)	HbA1c ≤ 7%		− 147.22 ± 113.79	− 97.80 ± 127.41	− 109.44 ± 107.64	− 106.98 ± 126.22
	HbA1c > 7%		− 130.41 ± 124.50	− 80.96 ± 109.71	− 44.78 ± 148.89	− 84.35 ± 129.65
	*p* value		0.506	0.508	**0.019**	0.405
BCVA (logMAR)	HbA1c ≤ 7%	0.48 ± 0.33	0.45 ± 0.29	0.43 ± 0.26	0.51 ± 0.36	0.48 ± 0.28
	HbA1c > 7%	0.54 ± 0.27	0.46 ± 0.27	0.50 ± 0.29	0.55 ± 0.30	0.50 ± 0.33
	*p* value	0.368	0.827	0.224	0.583	0.746
Change in BCVA from baseline (logMAR)	HbA1c ≤ 7%		− 0.04 ± 0.20	− 0.05 ± 0.24	0.03 ± 0.26	− 0.00 ± 0.24
	HbA1c > 7%		− 0.08 ± 0.15	− 0.04 ± 0.17	0.01 ± 0.21	− 0.04 ± 0.26
	*p* value		0.238	0.782	0.700	0.470

CST = central subfield thickness; HbA1c = glycosylated hemoglobin; BCVA = best-corrected visual acuity; logMAR = logarithm of the minimum angle of resolution.

Continuous variables are reported as mean ± standard deviation. Significant values with *p* < 0.05 are in bold.

**Figure 2:**
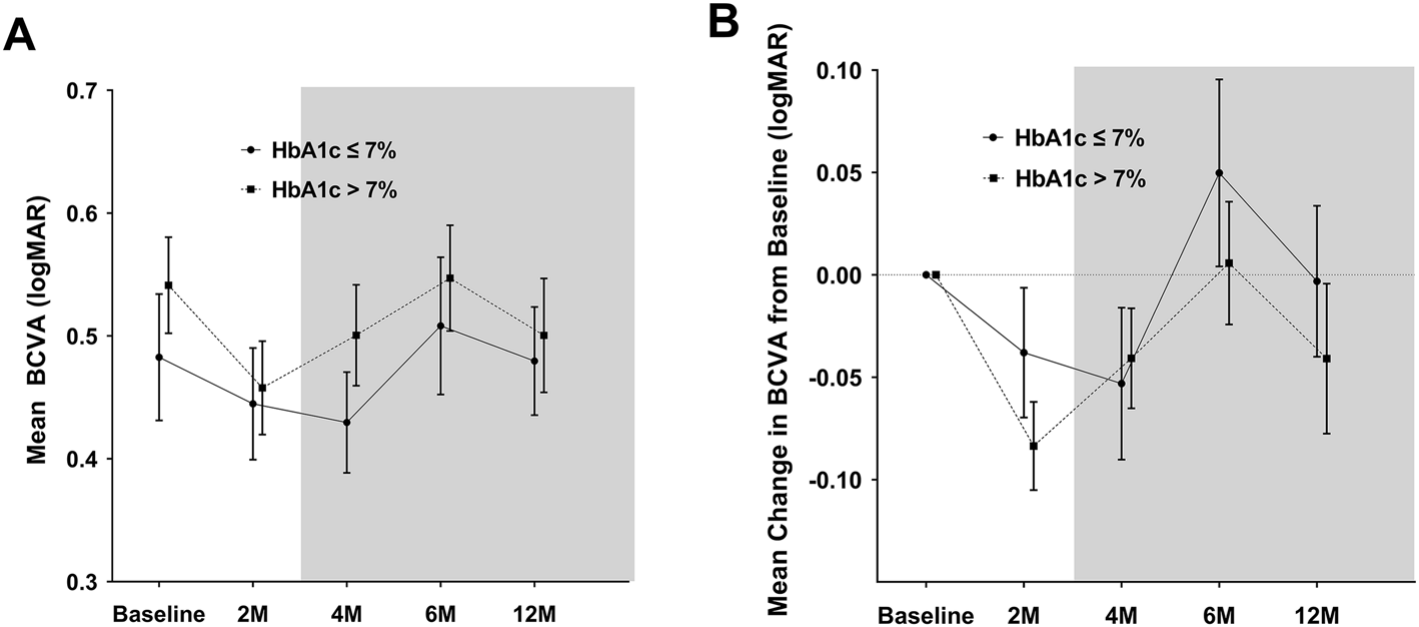
The mean BCVA (**A**) and the mean change in BCVA from baseline (**B**) through the follow-up periods in DEX implant-treated eyes with baseline HbA1c ≤ 7% compared with HbA1c > 7%. Circles and squares indicate means, and error bars represent ± 1 standard error. Asterisks denote significant difference (*p < 0.05, ** p < 0.01) between the two groups at each follow-up visit. BCVA = best-corrected visual acuity; logMAR = logarithm of the minimum angle of resolution; HbA1c = glycosylated hemoglobin.

**Figure 3:**
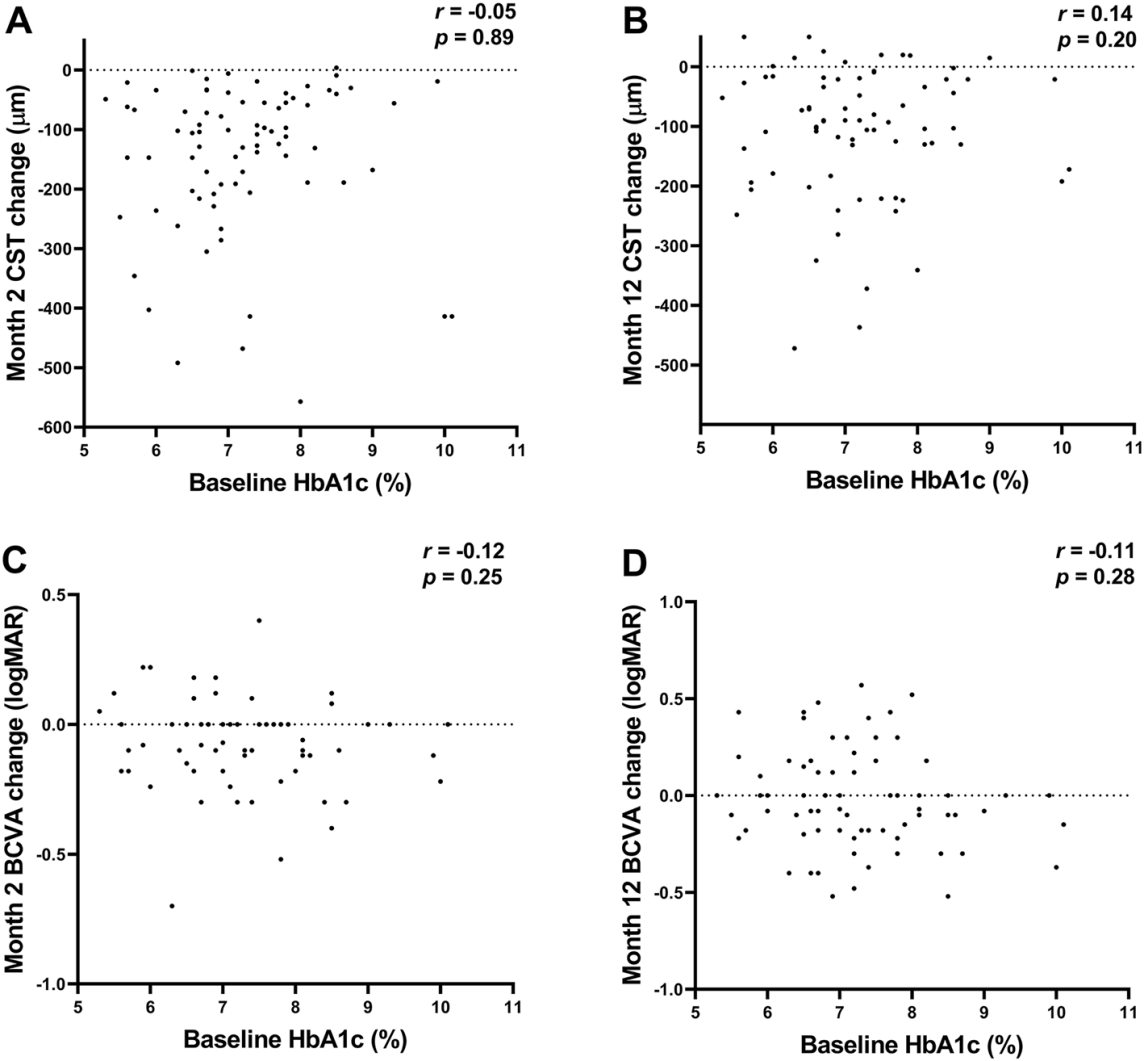
Scatterplots showing glycosylated hemoglobin (HbA1c) at baseline versus (**A**) change in central subfield thickness (CST) at month 2, (**B**) change in CST at month 12 (**C**) change in best-corrected visual acuity (BCVA) at month 2, and (**D**) change in BCVA at month 12. Pearson’s correlation coefficient (*r*) and *p* values for the slope of the regression line are noted.

The mean BCVA improved until 2 (patients with baseline HbA1c > 7%) to 4 months (patients with baseline HbA1c ≤ 7%) after DEX implant injection; however, there was a gradual deterioration until 6 months in both groups (Fig. 2). From baseline to 12 months, the mean BCVA and BCVA improvement were not significantly different between the two groups at any time. Because stratification by specific HbA1c may not accurately reflect the characteristics of the patient population, the association between change in CST and BCVA according to baseline HbA1c was further assessed. No significant associations were found at either month 2, when the pure effect of the DEX implant could be expected, or at month 12, the final follow-up period (Fig. 3).

The mean time to retreatment after DEX implant injection was 4.34 ± 4.56 months in patients with baseline HbA1c ≤ 7% and 6.04 ± 3.82 months in patients with baseline HbA1c > 7%, which was not significantly different but marginally higher in the latter group (*p* = 0.062). During the 12-month study period, the mean number of retreatments administered was 1.02 ± 1.15 in patients with baseline HbA1c ≤ 7% and 1.02 ± 0.80 in patients with baseline HbA1c > 7%, which was not significantly different (*p* = 0.985). The incidence of adverse events was not significantly different between the two subgroups (Table 1).

### Baseline characteristics: changes in HbA1c analysis

Among the 90 eyes, 27 (30.0%), 42 (46.7%), and 21 (23.3%) were included in the "improved", "stable" and "worsened" groups, respectively. The patient demographics and baseline characteristics are summarized in Table 3. The mean baseline HbA1c level was 7.91 ± 1.08% in the "improved" group, significantly higher than those of the "stable" (6.85 ± 0.73%) and "worsened" groups (6.83 ± 0.95%, *p* < 0.001). In the "improved" group, there were no patients who experienced an ‘early worsening’ in their HbA1c levels, defined as an increase of 0.5% or more from the baseline at the 2-month mark. The mean DM duration was 20.07 ± 9.17 years in the "improved" group, significantly higher than those of the "stable" (17.74 ± 9.44 years) and "worsened" groups (13.14 ± 6.78 years, *p* = 0.050). There was no significant difference in the proportion of patients taking SGLT2i between subgroups (*p* = 0.702). The mean baseline BCVA was logMAR 0.35 ± 0.21 in the "worsened" group, significantly better than those of the "improved" (logMAR 0.56 ± 0.31) and "stable" groups (logMAR 0.56 ± 0.31, *p* = 0.016). Age, sex, lens status, baseline CST, DR grading, and previous treatment for DME were not significantly different among the three subgroups.

**Table 3:** Patient demographics and baseline characteristics, and summary of retreatment: Change in glycosylated hemoglobin analysis.

Variable	Improved	Stable	Worsened	*p* value
Number of patients	27 (30.0%)	42 (46.7%)	21 (23.3%)	
HbA1c (%)	7.91 ± 1.08	6.85 ± 0.73	6.83 ± 0.95	< 0.001*
DM duration (years)	20.07 ± 9.17	17.74 ± 9.44	13.14 ± 6.78	0.050*
Use of SGLT2i	3 (11.1%)	3 (7.1%)	1 (4.8%)	0.702†
Age (years)	66.04 ± 7.10	65.33 ± 9.64	60.95 ± 9.89	0.117*
Men/Women	15/12	23/19	11/10	0.975†
RE/LE	12/15	23/19	13/8	0.470†
Phakia/Pseudophakia	16/11	24/18	15/6	0.533†
BCVA (logMAR)	0.56 ± 0.31	0.56 ± 0.31	0.35 ± 0.21	0.016*
CST (µm)	479.56 ± 136.53	436.17 ± 141.57	404.81 ± 84.04	0.134*
DR grade				0.131†
Moderate NPDR	2 (7.4%)	3 (7.1%)	3 (14.3%)	
Severe NPDR	13 (48.1%)	12 (28.6%)	3 (14.3%)	
PDR	12 (44.4%)	27 (64.3%)	15 (71.4%)	
Previous treatment for DME				0.454†
Treatment of naïve	10 (37.0%)	14 (26.4%)	10 (34.5%)	
Focal/grid laser	1 (3.7%)	6 (11.3%)	4 (14.8%)	
Anti-VEGF	6 (22.2%)	22 (41.5%)	12 (41.4%)	
Triamcinolone	2 (7.4%)	11 (20.8%)	3 (10.3%)	
Time to retreatment	5.30 ± 3.92	4.71 ± 3.42	6.33 ± 5.83	0.364*
Number of retreatments	1.04 ± 0.85	1.19 ± 1.09	0.67 ± 0.80	0.130*
Retreatment for DME				0.537†
Anti-VEGF	8 (29.6%)	14 (33.3%)	7 (33.3%)	
DEX implant	15 (55.6%)	19 (45.2%)	7 (33.3%)	
Triamcinolone	4 (14.8%)	5 (11.9%)	0	
Adverse event				
Cataract	3 (11.1%)	8 (19.0%)	3 (14.3%)	0.663†
Increased IOP	0	2 (4.8%)	1 (4.8%)	0.514†
Endophthalmitis	0	0	0	
Retinal detachment	0	0	0	

HbA1c = glycosylated hemoglobin; DM = diabetes mellitus; RE = right eye; LE = left eye; BCVA = best-corrected visual acuity; logMAR = logarithm of the minimum angle of resolution; CST = central subfield thickness; DR = diabetic retinopathy; NPDR = nonproliferative diabetic retinopathy; PDR = proliferative retinopathy; DME = diabetic macular edema; VEGF = vascular endothelial growth factor; DEX = dexamethasone; IO* p* = intraocular pressure; SGLT2i = Sodium-glucose co-transporter 2 inhibitor.

*Student’s *t*-test. †Chi-square test.

Continuous variables are reported as mean ± standard deviation. All other data are numbers (percentages). Significant values with *p* < 0.05 are in bold.

### Changes in HbA1c and clinical outcomes

**Figure 4:**
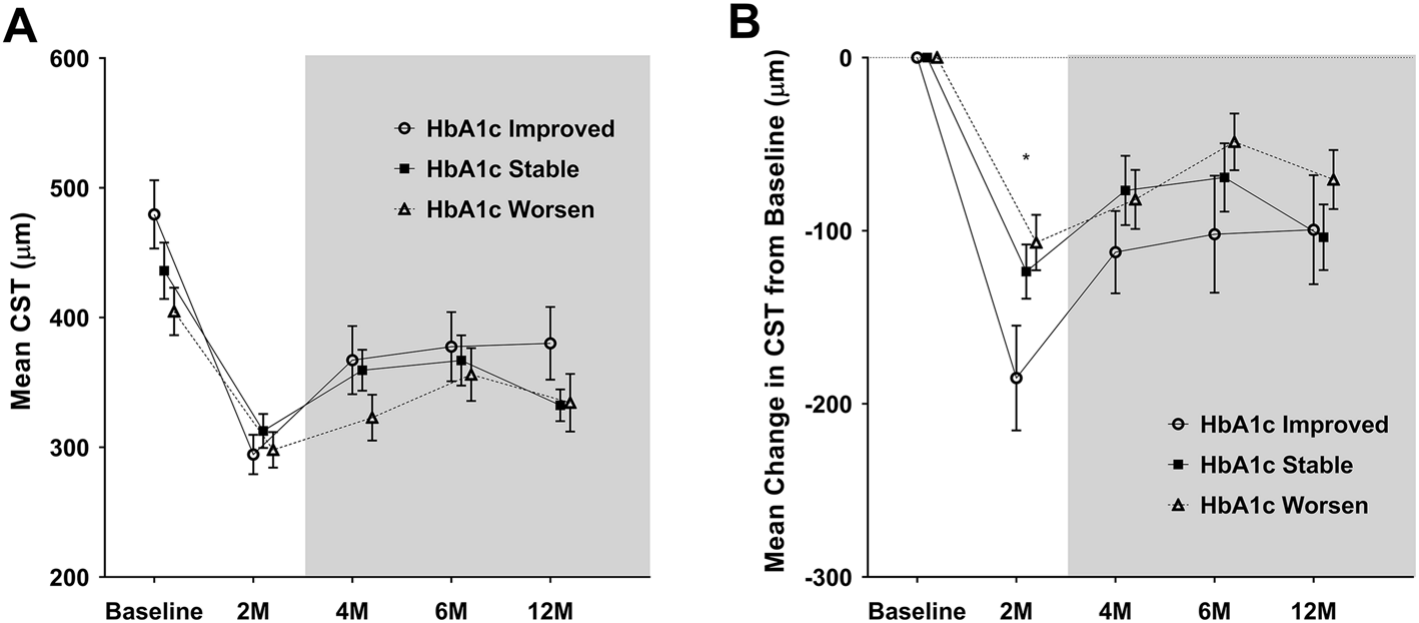
The mean CST (**A**) and the mean change in CST from baseline (**B**) through the follow-up periods in DEX implant-treated eyes according to the change in HbA1c levels. Circles and squares indicate means, and error bars represent ± 1 standard error. The gray shaded area is the time when retreatment was permitted. Asterisks denote significant difference (*p < 0.05, **p < 0.01) between the three groups at each follow-up visit. CST = central subfield thickness; HbA1c = glycosylated hemoglobin.

The anatomical and visual outcomes following changes in HbA1c levels are summarized in Table 4. At all time points, there was no significant difference in the mean CST between the three groups (Fig. 4). However, at 2 months, when the pure effect of the DEX implant was expected, the mean CST reduction from baseline was − 185.04 ± 157.59 µm in the "improved" group, significantly greater than those of the "stable" (− 123.52 ± 101.88 µm) and "worsened" groups (− 106.76 ± 73.47 µm, *p* = 0.042). There was no significant difference in the mean CST changes between the three groups from the time point thereafter until 12 months.

**Table 4:** Serial changes in mean CST, mean CST change, mean BCVA, and mean BCVA change were compared between 3 subgroups based on change in HbA1c levels.

Variable	Eyes	Baseline	2 months	4 months	6 months	12 months
CST (µm)	Improved	479.56 ± 136.53	294.52 ± 78.94	367.19 ± 136.52	377.59 ± 138.45	380.19 ± 145.71
	Stable	436.17 ± 141.57	312.64 ± 84.53	359.43 ± 102.67	366.98 ± 125.40	332.40 ± 79.50
	Worsened	404.81 ± 84.04	298.05 ± 62.98	322.90 ± 81.16	356.14 ± 93.63	334.43 ± 101.77
	*p* value	0.134	0.600	0.342	0.835	0.172
Change in CST (µm)	Improved		− 185.04 ± 157.59	− 112.37 ± 123.73	− 101.96 ± 175.73	− 99.37 ± 163.73
	Stable		− 123.52 ± 101.88	− 76.74 ± 129.82	− 69.19 ± 127.79	− 103.76 ± 122.89
	Worsened		− 106.76 ± 73.47	− 81.90 ± 78.15	− 48.67 ± 75.11	− 70.38 ± 78.39
	*p* value		**0.042**	0.455	0.381	0.610
BCVA (logMAR)	Improved	0.56 ± 0.31	0.49 ± 0.27	0.50 ± 0.33	0.52 ± 0.34	0.51 ± 0.37
	Stable	0.56 ± 0.31	0.51 ± 0.29	0.51 ± 0.25	0.62 ± 0.33	0.53 ± 0.26
	Worsened	0.35 ± 0.21	0.28 ± 0.17	0.33 ± 0.21	0.36 ± 0.23	0.39 ± 0.30
	*p* value	**0.016**	**0.004**	**0.036**	**0.009**	0.218
Change in BCVA (logMAR)	Improved		− 0.08 ± 0.17	− 0.06 ± 0.19	− 0.04 ± 0.20	− 0.05 ± 0.26
	Stable		− 0.05 ± 0.21	− 0.05 ± 0.24	0.06 ± 0.27	− 0.04 ± 0.22
	Worsened		− 0.07 ± 0.11	− 0.02 ± 0.13	0.01 ± 0.19	0.04 ± 0.28
	*p* value		0.806	0.767	0.237	0.421

CST = central subfield thickness; HbA1c = glycosylated hemoglobin; BCVA = best-corrected visual acuity; logMAR = logarithm of the minimum angle of resolution.

Continuous variables are reported as mean ± standard deviation. Significant values with *p* < 0.05 are in bold.

**Figure 5:**
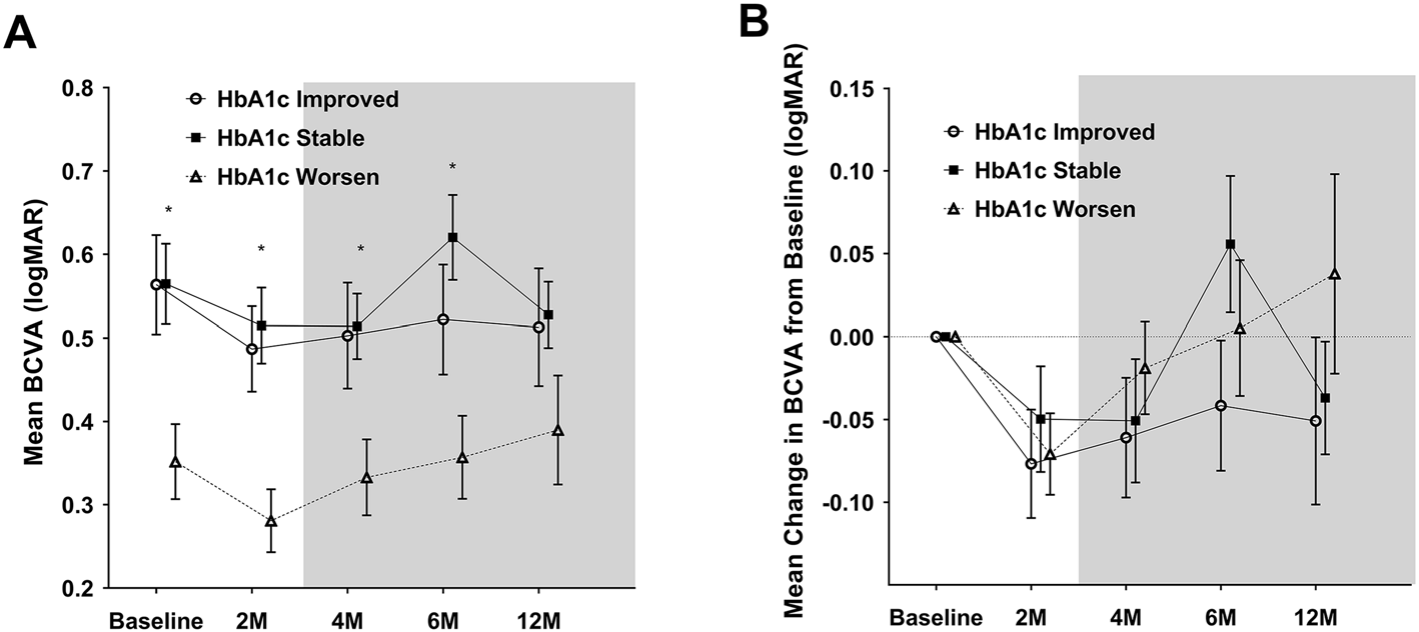
The mean BCVA (**A**) and the mean change in BCVA from baseline (**B**) through the follow-up periods in DEX implant-treated eyes according to the change in HbA1c levels. Circles and squares indicate means, and error bars represent ± 1 standard error. Asterisks denote significant difference (*p < 0.05, ** p < 0.01) between the three groups at each follow-up visit. BCVA = best-corrected visual acuity; logMAR = logarithm of the minimum angle of resolution; HbA1c = glycosylated hemoglobin.

The mean BCVA in the "worsened" group was significantly better at baseline and at 2 (*p* = 0.004), 4 (*p* = 0.036), and 6 months (*p* = 0.009) than that in the two other groups, but it gradually decreased over time (Fig. 5). At 12 months, the mean BCVA was not significantly different between the three groups. From baseline to 12 months, changes in mean BCVA were not significantly different among the three groups at any time point.

The mean time to retreatment after DEX implant injection was 5.30 ± 3.92, 4.71 ± 3.42, and 6.33 ± 5.83 months in the "improved," "stable," and "worsened" groups, respectively, which was not significantly different between the three groups (*p* = 0.364). During the 12-month study period, the mean numbers of retreatment administered were 1.04 ± 0.85, 1.19 ± 1.09, and 0.67 ± 0.80 in the "improved," "stable," and "worsened" groups, respectively, which were not significantly different between three groups (*p* = 0.130). The incidence of adverse events was not significantly different between the three subgroups (Table 3).

## Discussion

The study aimed to investigate the influence of serum baseline and the changes in HbA1c levels over 12 months on the treatment outcomes in patients with DME treated with intravitreal DEX implants. The findings can be summarized as follows: (1) Regardless of the baseline HbA1c levels in patients with DME, a significant reduction in CST and improvement in BCVA was observed 2 months after DEX implant injection; (2) According to the glycemic control levels, CST reduction in the "improved" group was greater at 2 months, but there was no significant difference at 12 months; and (3) The retreatment interval and number of additional injections did not differ according to the baseline or changes in HbA1c levels. This suggests that local alterations in the inflammation milieu may have a potentially stronger impact on DME treatment outcomes, highlighting the importance of considering local factors in DME treatment.

The relationship between HbA1c levels and risk for the development and progression of DR is well-established. The Diabetes Control and Complications Trial (DCCT)^11^ demonstrated that the risk of DR progression increases with higher HbA1c screenings and longer diabetes duration. The Epidemiology of Diabetes Interventions and Complications (EDIC) trial^12^ revealed that intensive therapy with low HbA1c levels could significantly lower the development of DME and the risk of DR progression. Do et al.^13^ reported that patients with type 2 diabetes and persistent DME had higher HbA1c levels than patients with resolved DME at the time of their disease. Another study showed that the incidence of DME is related to higher baseline HbA1c levels (hazard ratio, 1.17)^14^. However, the effect of HbA1c on the response to various DME treatment modalities, especially DEX implants, remains unclear.

There is conflicting evidence regarding the relationship between HbA1c levels and the response to anti-VEGF treatment for DME. Ozturk et al.^15^ found that baseline HbA1c was negatively correlated with CST changes, but there was no relationship between the baseline HbA1c level and BCVA improvement after a single intravitreal injection of ranibizumab. Similar findings from 124 patients were reported by Matsuda et al.^16^, wherein patients with HbA1c ≤ 7% achieved better final BCVA and greater CST reductions after 1 year of bevacizumab therapy. In addition, DRCR.net Protocol T^17^ reported that for each 1% elevation in HbA1c, there was a decrease of 1 letter in BCVA and a reduction of 0.5 letters in the BCVA area under curve analyses. However, in a post hoc analysis of ranibizumab-treated patients from the RIDE/RISE trials^18^, patients treated with monthly intravitreal ranibizumab showed improvements in BCVA and DR severity score and a CST reduction, independent of their baseline HbA1c or changed HbA1c levels. Similarly in a post hoc analysis of aflibercept-treated patients from the VISTA/VIVID trials^19^, mean BCVA improvement from baseline was independent of the baseline HbA1c level at 52 weeks but dependent at 100 weeks. The mean CST reduction from baseline was independent of baseline HbA1c at 52 and 100 weeks. Recently, in a prospective study of 35 patients, Wong et al.^20^ identified that good glycemic control (HbA1c ≤ 7%) in the period preceding anti-VEGF treatment caused greater reduction in CST, but the changes in BCVA after treatment did not correlate with the systemic factors tested.

Regarding the correlation between glycemic control and response to the DEX implant, the retrospective study of Pacella et al.^21^ reported that 2 of 19 eyes required retreatment, both of which had HbA1c > 7%, suggesting that worsening of the patient’s metabolic state caused a DME recurrence. Consistent with the current study’s observations, the multicenter retrospective cohort of the ARTES study group^22^ reported that the percentage of patients gaining visual acuity, anatomical outcomes, or the number of injections did not differ significantly between the controlled and uncontrolled patients (HbA1c ≥ 8%) with diabetes.

The current study noted significant CST reduction and BCVA improvement 2 months after DEX implant injection, regardless of baseline HbA1c. The analysis revealed a more significant increment in the mean CST at 6 months in patients with baseline HbA1c > 7%. This could be because the time taken to the first retreatment after DEX implant injection was 6.04 months in this group, which was later than the 4.34 months taken in patients with baseline HbA1c ≤ 7%, although the difference was not statistically significant. In the analysis of the changes in HbA1c, the "worsened" group showed a lower HbA1c level and better BCVA at baseline than the "improved" group. However, during the study period, the "worsened" group’s BCVA gradually deteriorated while losing glucose control. The "improved" group had greater CST reduction at 2 months; this may be because patients with more severe DME were included in this group. In relation to these findings, Oshima et al.^23^ have reported that the initiation of treatment for DME can enhance motivation for glycemic control, leading to an improvement in HbA1c levels. This effect was notably more significant in groups with higher baseline HbA1c levels (> 7.2%). Correspondingly, in our study, patients with HbA1c levels exceeding 7.0% were significantly more likely to be categorized in the improved group (*p* = 0.002).

The "worsened" group had a significantly better mean BCVA until the 6 months; however, this could be due to the significantly better baseline mean BCVA in this group. Rather, in the "worsened" group, vision gradually deteriorated during the treatment period; hence there was no significant difference in the final BCVA between the three groups. The changes in mean BCVA also did not differ between the three groups at any time point.

Systemic glycemic control may play a role in DME treatment outcomes, but it's challenging to prove a consistent relationship between one factor and treatment outcome, given the complex nature of DME pathogenesis. The current finding of no significant difference between systemic glycemic control and response to DEX implant treatment in DME has important clinical significance. It suggests that local treatment, such as DEX implant injection, should not be delayed because of uncontrolled systemic glucose levels in patients who have already developed DME. It also suggests that local alterations in the inflammation milieu may have a potentially stronger impact on DME treatment outcomes, while it is not reasonable to draw conclusions from this study because there are many confounding factors other than glycemic control.

Our study has several limitations, including possible selection bias due to its retrospective nature, a small sample size, and short follow-up periods. Due to the small number of patients, we were unable to perform propensity score matching according to the baseline HbA1c and DM duration on outcomes in the analysis by changes in HbA1c over time. Nevertheless, our case series is the largest study on the effect of systemic glucose control on DME treatment with DEX implants. Further studies with larger patient numbers and longer follow-up durations are needed to confirm our results. Moreover, DME is a complex condition that depends on various factors such as blood pressure, cholesterol, obesity, genetics, inflammation, VEGF milieu, and other systemic and genetic factors. These could not be controlled for in this retrospective study.

In conclusion, our study demonstrated that the benefit of DEX implant treatment in patients with DME was independent of their baseline HbA1c or change in HbA1c. Systemic glycemic control is still important. However, there should be no hesitation in initiating local treatment for DME on the grounds that systemic blood glucose control should precede it.

## Methods

### Participants

This study was performed at the Seoul National University Hospital (SNUH) in Korea, complying with the tenets of the Declaration of Helsinki. It was approved by the Institutional Review Board of SNUH (IRB approval number: 2003-092-1109). We retrospectively reviewed the medical records of patients who underwent intravitreal DEX implant injections for DME treatment between January 2013 and December 2020. Institutional Review Board of the SNUH waived the need for written informed consent from the participants, because of the study’s retrospective design.

The inclusion criteria were as follows: (1) center-involving DME (central subfield thickness [CST] ≥ 300 µm) treated with DEX implant; (2) serum HbA1c measurements at the time of DEX implant injection and after 1 year; and (3) a follow-up period of more than 1 year after DEX implant injection. The exclusion criteria were as follows: (1) pre-existing macular pathology such as age-related macular degeneration, macular hole, or vitreoretinal interface diseases such as macular pucker or vitreomacular traction syndrome; (2) previous treatment within 3 months prior to the DEX implant injection; (3) advanced glaucoma; (4) history of ocular trauma; (5) prior intraocular surgery except for non-complicated cataract surgery; and (6) significant media opacity that would considerably disturb optical coherence tomography (OCT) image acquisition.

At least 3 months after DEX implant injection, patients who experienced an increase in CST and/or a decrease in visual acuity received additional anti-VEGF agents or DEX implants based on the clinician's discretion.

### Examination and data collection

**Figure 6:**
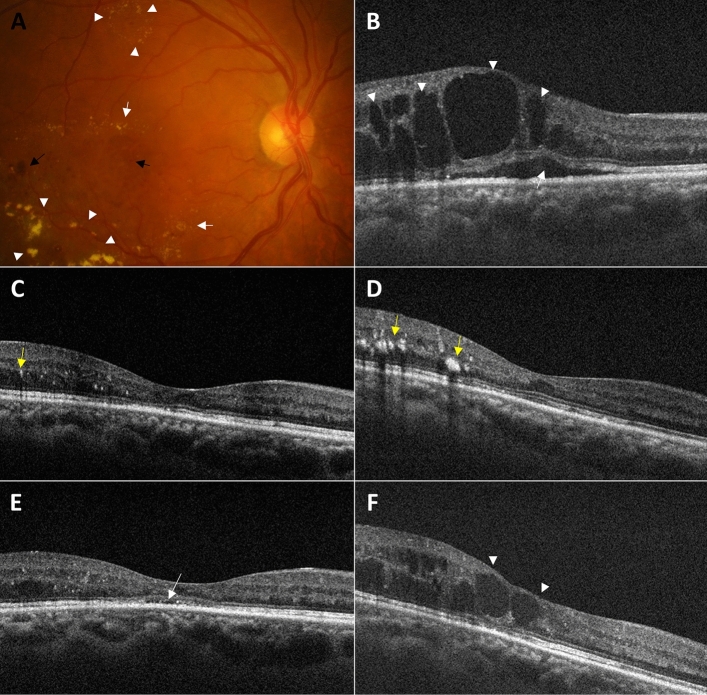
Representative diabetic macular edema case of a 61-year-old man with a baseline HbA1c of 7.9% and in the "improved" group. (**A**) Baseline fundus photography showing retinal hemorrhage (black arrow), hard exudates (white arrow), and circinate exudates (white arrowhead). (**B**) Baseline optical coherence tomography (OCT) revealing subretinal fluid (white arrow) and intraretinal fluid (white arrowhead). (**C**, **D**) OCT images showing hyperreflective hard exudates (yellow arrow) and a stable macula without fluid at 2 and 4 months after DEX implant injection, respectively. (**E**) OCT showing a subtle recurrence of subretinal fluid (white arrow) at 6 months after DEX implant injection. (**F**) OCT indicating a recurrence of intraretinal fluid (white arrowhead) at 12 months after DEX implant injection.

All patients underwent comprehensive ophthalmologic examination, including BCVA, slit-lamp, and dilated fundus examination. Spectral-domain OCT images were obtained using either a Heidelberg Spectralis (Heidelberg Engineering, Heidelberg, Germany) or Zeiss Cirrus (Cirrus 4000; Carl Zeiss Meditec, Dublin, CA). After DEX implant injection, BCVA measurement, tonometry, slit-lamp biomicroscopy, dilated fundus examination, and OCT were repeated at each follow-up visit. CST was defined as the mean retinal thickness in a 1-mm diameter circular zone centered on the fovea. BCVA measurements were converted to logarithm of the minimum angle of resolution (logMAR) units for statistical analyses. Considering the potential impact of SGLT2i on reducing DME^24^, an evaluation of SGLT2i usage was conducted at the baseline visit. Moreover, monitoring of any adverse events such as cataract formation, increased intraocular pressure, and development of endophthalmitis or retinal detachment were undertaken throughout the follow-up period. Figure 6 shows the fundoscopic findings and post-treatment serial OCT images of a representative case included in this study.

### Group classification according to glucose regulation

Serum HbA1c levels were measured at baseline and 12 months after DEX implant injection. According to the baseline HbA1c values, the patients were divided into two groups to assess the impact of baseline HbA1c on treatment outcomes. Patients with HbA1c ≤ 7.0% and > 7.0% were considered to have controlled diabetes and uncontrolled diabetes, respectively. This criterion was established following the 2021 American Diabetes Association position statement on the generally accepted threshold for diabetic control^25^. Furthermore, patients were divided into the following three subgroups on the basis of a ± 0.5% change in HbA1c levels from baseline to 12 months to investigate the influence of HbA1c changes on treatment outcomes—"improved" (baseline HbA1c decreased > 0.5% at 12 months), "stable" (change in baseline HbA1c within 0.5% at 12 months), and "worsened" (baseline HbA1c increased by > 0.5% at 12 months)^26^. This criterion was established following a clinically accepted threshold using which endocrinologists anticipate a meaningful response in improvement or worsening of glycemic status in clinical practice. The main outcome measures included the effect of glycemic control on the efficacy of DEX implants for DME regarding CST and BCVA.

### Statistical analysis

Statistical analyses were performed using the SPSS software for Windows (version 21.0; SPSS, Inc., Chicago, IL, USA). BCVA and CST in eyes treated with DEX implants were evaluated and compared among subgroups according to glucose regulation. Categorical variables were analyzed using the chi-squared test or Fisher's exact test. Continuous variables were compared using Student's *t*-test or Kruskal‒Wallis test, with the Mann‒Whitney U test as a post hoc test. Associations between baseline HbA1c and the changes in CST and BCVA were analyzed using the Pearson test. *p* values of less than 0.05 were considered statistically significant.

### Ethics approval

All procedures performed in studies involving human participants were in accordance with the ethical standards of the Seoul National University Hospital Institutional Review Board and with the 1964 Helsinki declaration and its later amendments or comparable ethical standards.

## Data Availability

The datasets used during the current study are available from the corresponding author on reasonable request.
